# Isoflurane Is More Deleterious to Developing Brain Than Desflurane: The Role of the Akt/GSK3**β** Signaling Pathway

**DOI:** 10.1155/2016/7919640

**Published:** 2016-02-01

**Authors:** Guorong Tao, Qingsheng Xue, Yan Luo, Guohui Li, Yimeng Xia, Buwei Yu

**Affiliations:** ^1^Department of Anesthesiology, Ruijin Hospital, Shanghai Jiaotong University School of Medicine, 197 Ruijin Er Road, Shanghai 200025, China; ^2^Department of Anesthesiology, Xinhua Hospital, Shanghai Jiaotong University School of Medicine, 1665 Kongjiang Road, Shanghai 200092, China

## Abstract

Demand is increasing for safer inhalational anesthetics for use in pediatric anesthesia. In this regard, researchers have debated whether isoflurane is more toxic to the developing brain than desflurane. In the present study, we compared the effects of postnatal exposure to isoflurane with those of desflurane on long-term cognitive performance and investigated the role of the Akt/GSK3*β* signaling pathway. Postnatal day 6 (P6) mice were exposed to either isoflurane or desflurane, after which the phosphorylation levels of Akt/GSK3*β* and learning and memory were assessed at P8 or P31. The phosphorylation levels of Akt/GSK3*β* and learning and memory were examined after intervention with lithium. We found that isoflurane, but not desflurane, impaired spatial learning and memory at P31. Accompanied by behavioral change, only isoflurane decreased p-Akt (ser473) and p-GSK3*β* (ser9) expressions, which led to GSK3*β* overactivation. Lithium prevented GSK3*β* overactivation and alleviated isoflurane-induced cognitive deficits. These results suggest that isoflurane is more likely to induce developmental neurotoxicity than desflurane in context of multiple exposures and that the Akt/GSK3*β* signaling pathway partly participates in this process. GSK3*β* inhibition might be an effective way to protect against developmental neurotoxicity.

## 1. Introduction

The developmental neurotoxicity of general anesthetics has aroused widespread concern in recent years. A large amount of evidence based on clinical retrospective studies indicates that children who experience multiple exposures to anesthesia before 3 years of age are more prone to learning disabilities when they mature [1, 2]. However, the contributions of specific anesthetics to this detrimental effect remain unclear. Inhalational anesthetics are a good choice for use in pediatric anesthesia due to their facile reversibility, their excellent analgesic effects, and their ability to induce muscle relaxation. Compared to other inhalational anesthetics, such as isoflurane and sevoflurane, desflurane is reported to have less serious effects with regard to neuroapoptosis and long-term cognitive decline [3, 4]. However, some studies have shown opposite results [5, 6]. To identify safer anesthetics for use as clinical anesthesia, the neurotoxicity of multiple exposures to various inhalational anesthetics and the mechanisms involved must be clarified.

Glycogen synthase kinase3*β* (GSK3*β*), a serine/threonine kinase, is highly expressed in the developing brain [7] and can be phosphorylated at serine 9 by Akt (protein kinase B), causing its functional suppression [8–10]. GSK3*β* plays important roles in normal brain development and memory formation via many mechanisms, including neuronal differentiation [11, 12], dendritic growth [13], and axon growth [14]. Additionally, GSK3*β* is involved in several pathophysiological processes, including apoptosis [15], autophagy [16], neural inflammation [17], and oxidative stress [17]. Overactivity of GSK3*β* contributes to ketamine-induced developmental neuroapoptosis [18] and cerebral ischemia-reperfusion impairment [19]; accordingly, inhibition of GSK3*β* might reverse such damage [18, 19].

In this study, the neurotoxic effects of multiple exposures of isoflurane and desflurane on the developing brain were compared. To identify the possible molecular targets involved, the potential role of the Akt/GSK3*β* signaling pathway was further investigated.

## 2. Methods

### 2.1. Animals

The animal treatment protocols utilized in this study were approved by the Ethics Committee for the Care and Use of Laboratory Animals of Shanghai Jiao Tong University. C57BL/6 mother mice and their postnatal day 6 (P6) pups were acquired from SLAC Laboratory Animal Co., Ltd. (Shanghai, China). All mice were housed at constant temperature (22°C) under a 12 : 12 h light-dark cycle with* ad libitum* access to food and water; the pups were reared by their mother mice.

### 2.2. Anesthesia

P6 mice (both genders) in the experimental groups were anesthetized with either 2% isoflurane or 8% desflurane balanced with 60% oxygen plus nitrogen. The doses of isoflurane and desflurane (0.70 to 0.74 MAC) were selected according to a previous study [5] and confirmed in our preliminary experiment. The treatment was applied for two hours per day for three consecutive days. Control groups received only 60% oxygen plus nitrogen for the same periods in similar chambers and conditions. The concentrations of inhalation anesthetics and oxygen were measured continuously at the chamber outlets using a gas analysis system (Datex-Ohmeda, Capnomac Ultima, Finland). During anesthesia, the rectally measured temperature of the mice were maintained at 37.0 ± 0.5°C using a heating plate. For the intervention studies, lithium chloride (100 mg/kg), a GSK3*β* inhibitor, was administered intraperitoneally to the pups 30 min before isoflurane or oxygen treatment. The pups in the control group were injected intraperitoneally with the same volume of saline. A total of 17 litters including 119 P6 mice were used in this study. To minimize the difference among different mother mice, we divided the same number of mice from one litter into each experiment group as far as possible. None of the mice died in the whole process of anesthesia.

### 2.3. Morris Water Maze Test

For behavioral tests, one group of mice receiving three times of anesthesia or control treatment were allowed to raise until P31 to assess spatial learning and memory using Morris water maze (MWM) test as described previously with some modifications [20]. Briefly, the P31 mice were trained for place trials and probe trials in a round steel tank (100 cm in diameter and 60 cm in depth) that contained a hidden 10 cm diameter platform. The place trials were performed four times daily for seven successive days. For each trial, the mice were allowed 90 s to find the platform in the tank and were permitted to remain on the platform for 15 s after finding it. Otherwise, the mice were gently guided to the platform and permitted to remain there for 15 s. Escape latencies were recorded to evaluate changes in spatial learning. For each mouse, the probe trial was performed 24 hours after finishing the last place trial on the seventh day. During the probe trial, the mice were allowed to swim for 90 s after removing them from the platform. Platform crossing times were measured to evaluate changes in spatial memory for each group.

### 2.4. Fear Conditioning Test

The fear conditioning test (FCT) was performed as described previously with some modification [4, 21]. Five days after the MWM test, fear conditioning was investigated in various groups of mice at P42. During the training period, each mouse was allowed to explore the chamber freely for 180 s, after which a 60 s 2 Hz pulsating tone (80 dB, 1,500 Hz) was imposed followed by a mild foot shock (0.8 mA for 0.5 s). During the interval between each session, the chamber was cleaned with 70% alcohol to eliminate any odors. Fear conditioning memory was evaluated 48 hours after training. In the contextual test, each mouse was reexposed to the same chamber and allowed to explore for 180 s, followed by a 180 s rest (within the chamber) without a tone, and finally allowed to recover for 30 s. In the tone test, each mouse was allowed to explore a modified chamber comprising a plastic floor and cardboard walls for 180 s; during the following 180 s, a tone was sounded; subsequently, the mouse was allowed to recover for 30 s. The freezing time of each mouse was recorded during the second 180 s period using Observer software (Any-Maze, Stoelting).

### 2.5. Western Blot Analysis

For mechanism studies, the hippocampi from another group of mice were harvested immediately after three times of anesthesia or control treatment and lysed in ice-cold RIPA buffer supplemented with PMSF and a protease and phosphatase inhibitor cocktail solution. The protein concentrations of the lysates were measured using a BCA assay kit (Pierce, Iselin, NJ, USA). The proteins were resolved using SDS-PAGE and then electrophoretically transferred onto polyvinylidene difluoride membranes (Millipore, Billerica, MA, USA). The membranes were blocked with 5% nonfat milk and subsequently incubated with primary rabbit anti-p-Akt (ser473), anti-Akt, anti-p-GSK3*β* (ser9), anti-GSK3*β* (1 : 1,000, Cell Signaling, Danvers, MA, USA), or mouse anti-*β*-actin (1 : 5,000, Sigma, St. Louis, MO, USA) antibodies with gentle agitation overnight at 4°C. After being washed with TBST buffer at room temperature (RT), The membranes were then incubated with horseradish peroxidase-labeled goat anti-rabbit (1 : 2,000) or goat anti-mouse (1 : 5,000) secondary antibodies for 1 h at RT. The protein bands were visualized using ImageQuant LAS 4000 minisystem (GE Healthcare Bio-Sciences, Pittsburgh, PA, USA). The signal intensity of bands was analyzed using ImageJ software.

### 2.6. Statistical Analysis

Data from western blot analyses, FCT, and place trials of the MWM tests were expressed as the means ± SEM. However, platform crossing time data (obtained during the probe trials of the MWM tests) were expressed as medians and interquartile ranges. Among-group differences in protein expression levels and freezing times pertaining to the FCT were analyzed using Student's *t*-test or one-way ANOVA followed by* post hoc* Bonferroni tests. Two-way repeated measures ANOVA were used to compare the difference of learning curves (based on escape latency) among groups in the MWM place trials.* Post hoc* Bonferroni tests were used to compare the difference in escape latency among groups in each day of MWM place trials. The Mann-Whitney test was used to calculate differences in platform crossing time among groups. *P* values of less than 0.05 (*∗*), 0.01 (*∗∗*), and 0.001 (*∗∗∗*) were considered statistically significant. Statistical analyses were performed using SPSS 18.0 software (SPSS Inc., Chicago, IL, USA). All graphs were plotted using PRISM 5 software (GraphPad, La Jolla, CA, USA) and Adobe Illustrator Artwork 16.0 software (Adobe Inc., San Jose, California, USA).

## 3. Results

### 3.1. Isoflurane, but Not Desflurane, Induced Impairment of Long-Term Learning and Memory

**Figure 1:**
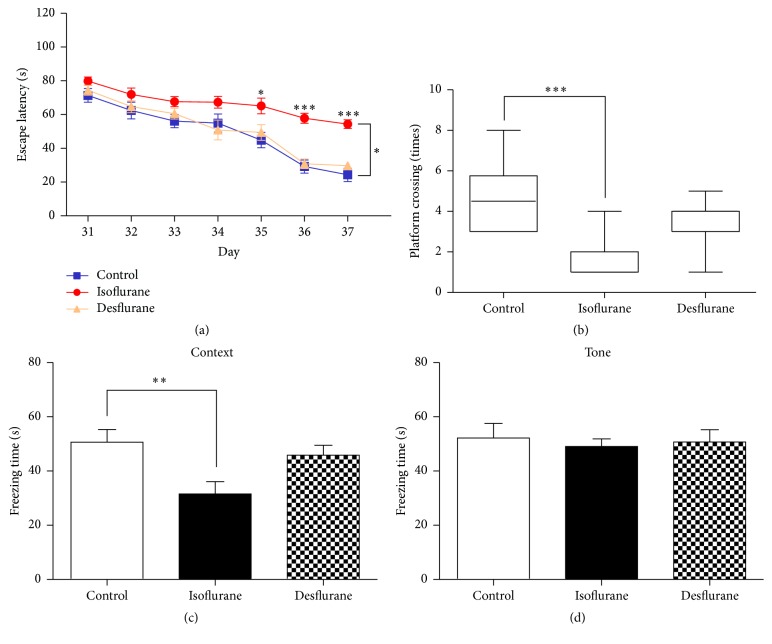
Isoflurane, but not desflurane, impaired learning and memory in young mice. (a) In place trials of MWM, escape latency in each group was recorded and analyzed. (b) In the probe trials of MWM, the platform crossing times in each group were recorded and analyzed. (c) Bar shows the freezing time in context trials of FCT. (d) Bar shows the freezing time in cue trials of FCT. The data in (a), (c), and (d) are presented as mean ± SEM, the data in (b) are presented as median and 95% confidence interval, ^*∗*^ *P* < 0.05, ^*∗∗*^ *P* < 0.01, and ^*∗∗∗*^ *P* < 0.001 compared with control group, *n* = 12-13 for each group, MWM = Morris water maze, and FCT = fear conditioning test.

First, we assessed the effect of three exposures to 2% isoflurane or 8% desflurane on long-term spatial learning and memory at P31 using the MWM. As shown in Figure 1(a), the escape latency times of mice treated with isoflurane for 2 h daily for 3 days were significantly higher than those recorded for mice that were treated only with oxygen (*F* = 2.734, *P* = 0.0153, two-way ANOVA with repeated measurements). However, no significant differences in escape latency (in MWM tests) were observed between mice treated with desflurane and mice treated only with oxygen (*F* = 0.3291, *P* = 0.9206, two-way ANOVA with repeated measurements) (Figure 1(a)). In the probe tests, the platform crossing times obtained for the isoflurane group (Figure 1(b), *P* = 0.0001), but not the desflurane group (Figure 1(b), *P* = 0.1294), were significantly lower than those obtained for the control group. These results indicate that multiple exposures of isoflurane are more potent than multiple exposures of desflurane at inducing long-term spatial learning and memory impairment.

The fear conditioning test (FCT) was used to evaluate the learning and memory of mice in response to treatment with isoflurane or desflurane. These FCT included contextual fear conditioning tests and tone fear conditioning tests, which estimated hippocampus-dependent and hippocampus-independent fear memory, respectively. One-way ANOVA showed that three exposures to isoflurane (Figure 1(c), *P* < 0.01), but not desflurane (Figure 1(c), *P* > 0.05), significantly decreased the freezing times recorded during FCT that were conducted 48 hours after training. However, no significant differences in freezing times were observed in FCT tone tests among the isoflurane, desflurane, and control groups (Figure 1(d), *P* > 0.05). These results show that isoflurane mainly impaired hippocampus-dependent memory, consistent with the MWM test results.

### 3.2. Isoflurane, but Not Desflurane, Decreased the Phosphorylation of Akt and GSK3*β*

**Figure 2:**
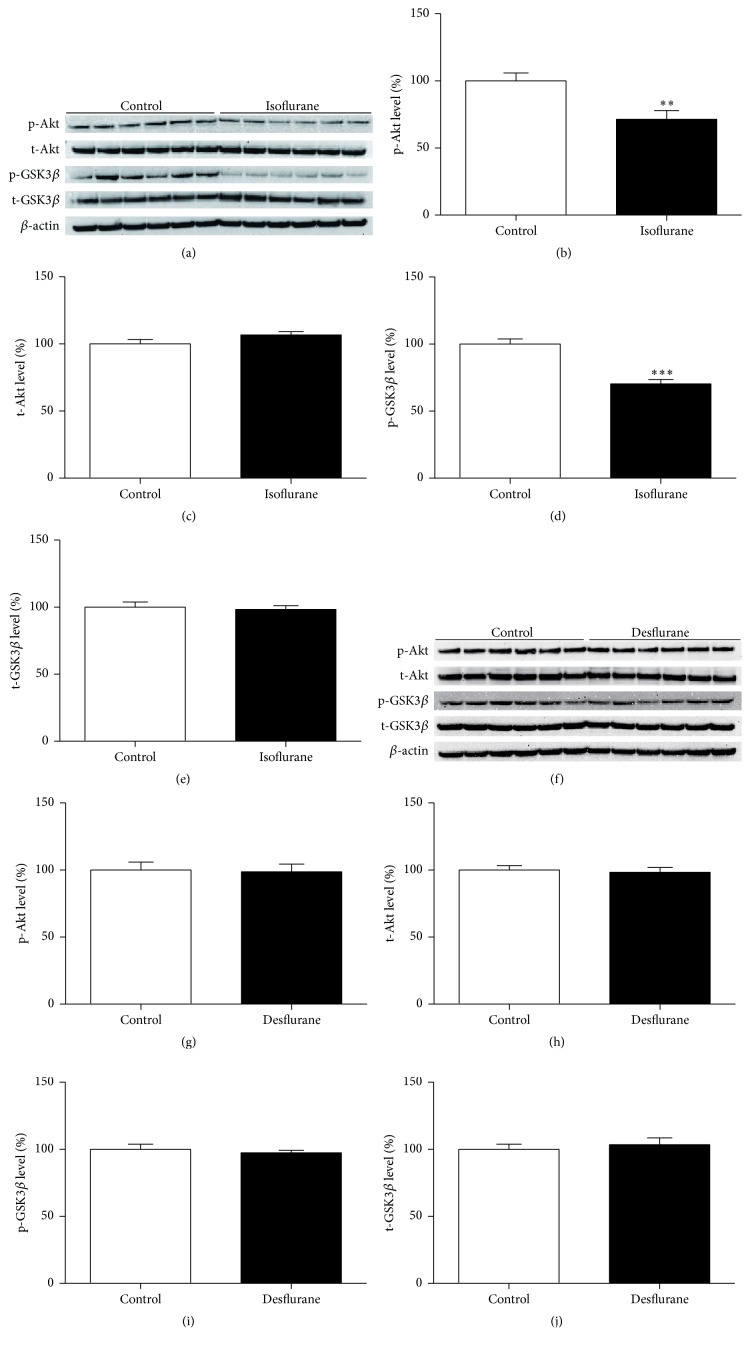
Isoflurane, but not desflurane, decreased phosphorylation of Akt and GSK3*β* in mice hippocampus. (a) Immediately after multiple anesthesia with 2% isoflurane, p-Akt (ser473), t-Akt, p-GSK3*β* (ser9), t-GSK3*β*, and *β*-actin in the total protein extracted from mice hippocampus tissues were determined by western blot analysis. (b) The histogram shows p-Akt (ser473)/*β*-actin ratio in (a). (c) The histogram shows t-Akt/*β*-actin ratio in (a). (d) The histogram shows p-GSK3*β*(ser9)/*β*-actin ratio in (a). (e) The histogram shows t-GSK3*β*/*β*-actin ratio in (a). (f) Immediately after multiple anesthesia with 8% desflurane, p-Akt (ser473), t-Akt, p-GSK3*β* (ser9), t-GSK3*β*, and *β*-actin in the total protein extracted from mice hippocampus tissues were determined by western blot analysis. (g) The histogram shows p-Akt (ser473)/*β*-actin ratio in (f). (h) The histogram shows t-Akt/*β*-actin ratio in (f). (i) The histogram shows p-GSK3*β* (ser9)/*β*-actin ratio in (f). (j) The histogram shows t-GSK3*β*/*β*-actin ratio in (f). All data are presented as mean ± SEM, ^*∗∗*^ *P* < 0.01 and ^*∗∗∗*^ *P* < 0.001 compared with control group, and *n* = 6 for each group.

Because decreased Akt and GSK3*β* phosphorylation have been associated with learning and memory impairment [22–24], we investigated the effects of three exposures to 2% isoflurane and 8% desflurane on Akt and GSK3*β* phosphorylation levels in the hippocampus. Figures 2(a) and 2(f) showed the expression of p-Akt (ser473), total-Akt, p-GSK3*β* (ser9), total-GSK3*β*, and *β*-actin in groups of isoflurane, desflurane, or control treatments. Student's *t*-test analyses showed that three exposures to isoflurane, but not desflurane, significantly decreased the expression of p-Akt (ser473) (Figure 2(b), *P* < 0.01) (Figure 2(g), *P* > 0.05) and p-GSK3*β* (ser9) (Figure 2(d), *P* < 0.001) (Figure 2(i), *P* > 0.05) in mouse hippocampus tissue compared to control conditions. However, neither isoflurane nor desflurane significantly affected total-Akt (Figure 2(c), *P* > 0.05) (Figure 2(h), *P* > 0.05) or total-GSK3*β* expression levels (Figure 2(e), *P* > 0.05) (Figure 2(j), *P* > 0.05).

### 3.3. Lithium Pretreatment Attenuated Long-Term Learning and Memory Impairment Caused by Postnatal Isoflurane Exposure

**Figure 3:**
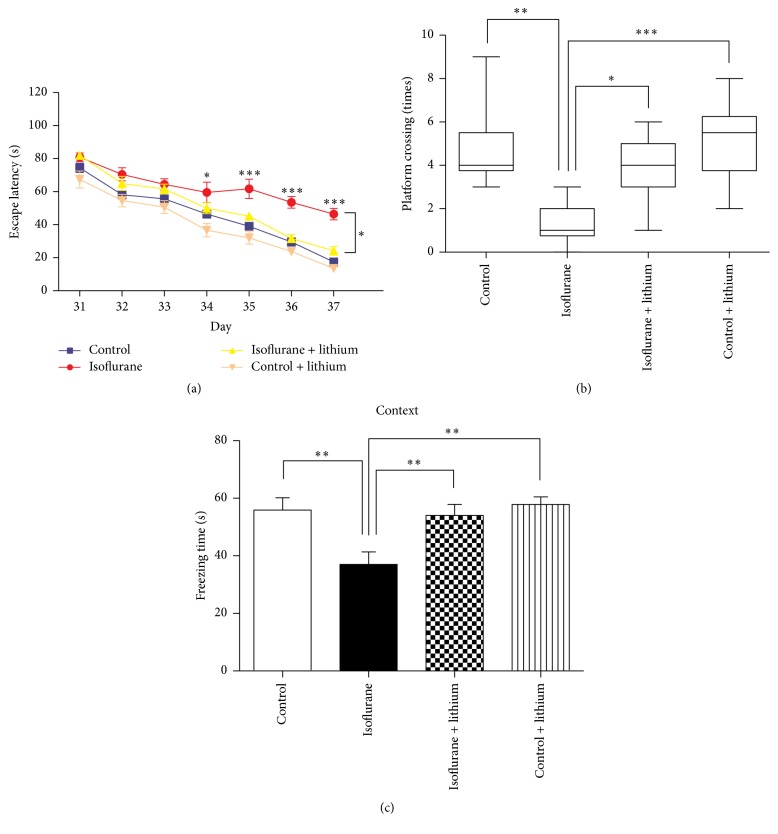
Lithium pretreatment attenuated learning and memory impairment of mice treated with isoflurane. (a) The escape latency to find the submerged platform in each group was calculated. (b) In probe trials of MWM, the platform crossing times in each group were calculated and analyzed. (c) In the context trials of FCT, the freezing time in each group 48 hours after training was recorded. The data in (a) and (c) are presented as mean ± SEM. The data in (b) are presented as median and 95% confidence interval. ^*∗*^ *P* < 0.05, ^*∗∗*^ *P* < 0.01, and ^*∗∗∗*^ *P* < 0.001; *n* = 10 in each group. MWM = Morris water maze. FCT = fear conditioning test.

Lithium, an inhibitor of GSK3*β*, exhibits neuroprotective effects in various models [25–27]. Therefore, we investigated whether lithium pretreatment can reverse isoflurane-induced learning and memory impairment. The administration of lithium (100 mg/kg, administered intraperitoneally 30 min before each isoflurane anesthesia treatment) effectively decreased escape latency during place trials (two-way ANOVA, *F* = 1.741, *P* = 0.0332) (Figure 3(a)). Furthermore, compared to the saline-treated group, lithium attenuated isoflurane-induced decreases in platform crossing times determined during probe trials (Figure 3(b), *P* = 0.0001). Lithium exhibited similar neuroprotective effects against isoflurane during FCT (based on freezing time) (Figure 3(c), *P* < 0.01). These MWM and FCT results suggest that lithium might exhibit protective effects against isoflurane-induced neurotoxicity.

### 3.4. Lithium Restored Decreased GSK3*β* and Akt Phosphorylation in the Hippocampal Tissue of Isoflurane-Treated Mice

**Figure 4:**
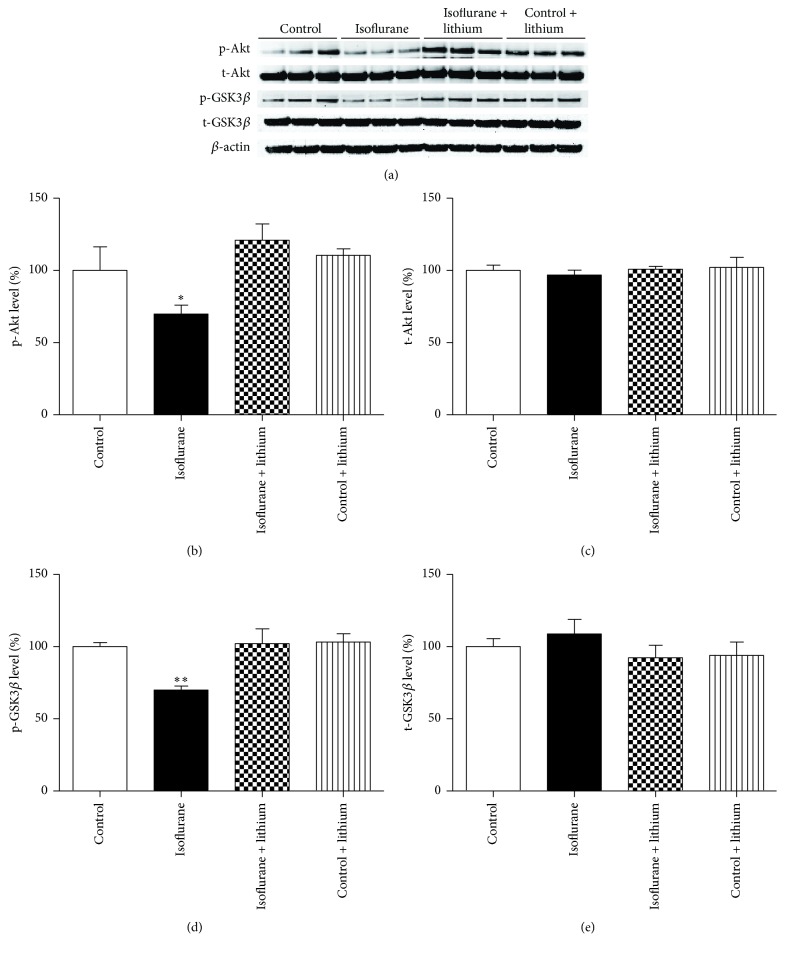
Lithium attenuated isoflurane-induced decrease in Akt and GSK3*β* phosphorylation in mice hippocampus. (a) Lithium (100 mg/kg) was administered intraperitoneally 30 min before every treatment with a 2 h 2% isoflurane daily for 3 days. Immediately after the last exposure, the expression levels of p-Akt, t-Akt, p-GSK3*β*, and t-GSK3*β* in the hippocampus tissues of mice were determined by Western blot analysis. (b) Bar shows the levels of p-Akt/*β*-actin ratio in (a). (c) Bar shows the levels of t-Akt/*β*-actin ratio in (a). (d) Bar shows the levels of p-GSK3*β*/*β*-actin ratio in (a). (e) Bar shows the levels of t-GSK3*β*/*β*-actin ratio in (a). All data are presented as mean ± SEM, ^*∗*^ *P* < 0.05 and ^*∗∗*^ *P* < 0.01 compared with control group; *n* = 6 for each group.

To elucidate the mechanism underlying the neuroprotective effects of lithium, we investigated whether lithium could reverse the decrease in the levels of p-GSK3*β* (ser9) caused by three exposures to isoflurane.  Figure 4(a) showed the expression of p-Akt (ser473), total-Akt, p-GSK3*β* (ser9), total-GSK3*β*, and *β*-actin in groups of control and isoflurane and isoflurane + lithium and control + lithium mice. One-way ANOVA showed that lithium treatment prior to isoflurane anesthesia significantly reversed isoflurane-induced decreases in the levels of p-Akt (ser473) (Figure 4(b), *P* < 0.05) and p-GSK3*β* (ser9) (Figure 4(d), *P* < 0.01). However, neither lithium nor isoflurane exhibited significant effects on total-Akt (Figure 4(c), *P* > 0.05) and total-GSK3*β* (Figure 4(e), *P* > 0.05) expression in the hippocampi investigated. These results suggested that the effects of lithium on reversing isoflurane neurotoxicity result, at least partly, from increased Akt and GSK3*β* phosphorylation.

## 4. Discussion

Three exposures to isoflurane were found more likely to induce long-term memory impairment than three exposures of desflurane. Further investigation indicated that the Akt/GSK3*β* signaling pathway was involved in the neurotoxicity caused by multiple exposures to isoflurane. Interestingly, lithium potently attenuated the neurotoxicity of isoflurane by increasing GSK3*β* phosphorylation. These results suggested that the Akt/GSK3*β* signaling pathway might contribute to isoflurane-induced neurotoxicity in the developing brain.

As shown in Figure 1, we found that multiple exposures of postnatal day 6 mice to clinically relevant concentrations of isoflurane, but not desflurane, induced learning and memory impairment as the mice matured. In the molecular structure, isoflurane and desflurane both contain a fluorinated ethyl group and a difluoromethyl group [28]. However, isoflurane contains a chlorine atom attached to the ethyl group, which is replaced by a fluorine atom in desflurane [29]. Whether this structure difference between isoflurane and desflurane contributed to their distinct developmental neurotoxicity should be clarified in further experiments. The findings in our study are consistent with previous animal and human studies of neuronal injury resulting from inhalational anesthetics [4]. Single exposure to isoflurane can induce more serious cell injuries than single exposure to desflurane due to its disruption of intracellular calcium homeostasis [30] and mitochondrial function [4]. A recent pilot study indicates that patients receiving isoflurane anesthesia have a higher morbidity for postoperative cognitive decline than patients receiving desflurane [31].

The present study showed that multiple anesthetic exposures to isoflurane, but not desflurane, decreased the phosphorylation of hippocampal GSK3*β* at ser9 (Figure 2), suggesting that GSK3*β* is more likely to be activated after isoflurane exposure. This result is consistent with studies of other inhalational anesthetics, such as sevoflurane. Sevoflurane induces developmental neurotoxicity by activating GSK3*β* in vitro and in vivo [32, 33]. Recent experimental evidence indicates that inhalational anesthetics induce long-term cognitive decline partly by triggering neuroapoptosis, oxidative stress, and neuroinflammation in the developing brain [20, 34–38]. GSK3*β* promotes apoptosis by upregulating proapoptotic proteins (such as Bax, Bad, and P53) and by downregulating antiapoptotic proteins (such as Bcl-2 and survivin) [39]. Furthermore, under conditions of oxidative stress, GSK3*β* translocates to the mitochondria and promotes mPTP opening, thereby releasing caspase-3 and initiating apoptosis initiation [40]. In addition, GSK3*β* mediates the release of proinflammatory cytokines, including IL-1*β*, TNF-*α*, and IL-8 and anti-inflammatory cytokines (such as IL-10) from cortical glia and brain endothelial cells [41]. Inhibition of GSK3*β* contributes to stabilization of the blood-brain barrier and reduced proinflammatory cytokines release, finally leading to decreased neuroinflammation responses [42]. However, whether GSK3*β* activation participates in isoflurane-induced apoptosis, oxidative stress, and neuroinflammation requires further clarification. We plan to investigate this issue further using selective pharmacological inhibitors or gene knockdown technology.

Akt is a serine/threonine-specific protein kinase that plays a key role in normal brain development, involving the proliferation [43, 44] and differentiation and migration [32] of neural precursors. Moreover, Akt is an upstream regulator kinase of GSK3*β* [45]. As shown in Figure 2, multiple exposures to isoflurane, not desflurane, were found to reduce p-Akt (ser473) and p-GSK3*β* (ser9) expression levels in the hippocampi of young mice, suggesting that inconsistencies in Akt/GSK3*β* activation might be associated with the different effects of isoflurane and desflurane on learning and memory in later age. Previous studies have demonstrated that isoflurane-induced memory decline is paralleled by decreased progenitor proliferation and reduced neurogenesis and synaptogenesis impairment in the developing brain [46–49]. However, whether the Akt/GSK3*β* signaling pathway is responsible for the above-described injuries in our model remains unclear. The Akt/GSK3*β* signaling pathway is critical for the neurogenesis, synaptogenesis, and synaptic plasticity associated with memory in rats, regardless of age. Inhibition of GSK3*β* activation promotes synaptogenesis in Drosophila and mammalian neurons [50]. Increasing GSK3*β* activity abolishes neurogenesis in the dentate gyrus of the hippocampus as well as memory induced by environmental enrichment [51]. GSK3*β* activation is involved in the impairment of memory consolidation caused by sevoflurane in adult rats [52].

Lithium, a GSK3*β* inhibitor, can protect neurons after hypoxia [53], ischemia [54], ethanol exposure [55], and irradiation [56]. In the present study, lithium was found to restore cognitive impairment (Figure 3) and reduce the phosphorylation of GSK3*β* (Figure 4) by multiple isoflurane exposures. Our findings are consistent with previous studies that reported learning and memory improvement after lithium administration [57, 58]. Lithium might exert its protective effects by preventing neuroapoptosis and restoring synaptic plasticity via increased GSK3*β* phosphorylation [18, 25, 57].

In conclusion, anesthesia treatment with 2% isoflurane, but not 8% desflurane, for two hours daily over three consecutive days in young (P6) mice impaired learning and memory possibly through Akt/GSK3*β* signaling pathway. These results partly illustrate the mechanisms by which isoflurane induces neurotoxicity in the developing brain. Additionally, the finding of lithium's effects against isoflurane neurotoxicity might offer a cue for routine anesthesia practice.
